# A biomechanical analysis of four anterior cervical techniques to treating multilevel cervical spondylotic myelopathy: a finite element study

**DOI:** 10.1186/s12891-021-04150-7

**Published:** 2021-03-15

**Authors:** Zhonghai Li, Hui Liu, Ming Yang, Wentao Zhang

**Affiliations:** 1 Department of Orthopaedics, First Affiliated Hospital of Dalian Medical University, Dalian, People’s Republic of China; 2 Key Laboratory of Molecular Mechanism for Repair and Remodeling of Orthopaedic Diseases, Liaoning Province, People’s Republic of China; 3 Seventh Medical Center of PLA General Hospital, Beijing, People’s Republic of China

**Keywords:** Multilevel cervical spondylotic myelopathy, Anterior cervical corpectomy and fusion, Anterior cervical discectomy and fusion, Biomechanics, Finite element analysis

## Abstract

**Background:**

The decision to treat multilevel cervical spondylotic myelopathy (MCSM) remains controversial. The purpose of this study is to compare the biomechanical characteristics of the intervertebral discs at the adjacent segments and internal fixation, and to provide scientific experimental evidence for surgical treatment of MCSM.

**Methods:**

An intact C2-C7 cervical spine model was developed and validated. Four additional models were developed from the fusion model, including multilevel anterior cervical discectomy and fusion (mACDF), anterior cervical corpectomy and fusion (ACCF), hybrid decompression and fusion (HDF), and mACDF with cage alone (mACDF-CA). Biomechanical characteristics on the plate and the disc of adjacent levels (C2/3, C6/7) were comparatively analyzed.

**Results:**

Of the four models, stress on the upper (C2/3) adjacent intervertebral disc was the lowest in the mACDF-CA group and highest in the ACCF group. Stress on the intervertebral discs at adjacent segments was higher for the upper C2/3 than the lower C6/7 intervertebral disc. In all models, the mACDF-CA group had the lowest stress on the intervertebral disc, while the ACCF group had the highest stress. In the three surgical models with titanium plate fixation (mACDF, ACCF, and HDF), the ACCF group had the highest stress at the titanium plate-screw interface, while the mACDF group had the lowest stress.

**Conclusion:**

Among the four anterior cervical reconstructive techniques for MCSM, mACDF-CA makes little effect on the adjacent disc stress, which might reduce the incidence of adjacent segment degeneration (ASD) after fusion. However, the accompanying risk of the increased incidence of cage subsidence should never be neglected.

## Background

Multilevel cervical spondylotic myelopathy (MCSM) refers to cervical spondylosis diagnosed by imaging with three or more levels of contiguous or noncontiguous cervical intervertebral disc degeneration and secondary changes, which causes compression on the dural sac and spinal cord, and which results in corresponding clinical manifestations. Owing to severe spinal cord compression in most cases, MCSM often requires surgery to relieve the compression. Consensus has currently been reached on the surgical management of CSM involving one or two mobile segments; however, controversy remains regarding the selection of surgical procedures for treatment of MCSM [1–3].

An anterior, posterior, or combined anterior-posterior approach can be employed according to the clinical situation and the experience of surgeons, and each approach has its unique advantages and disadvantages [3–12]. The anterior techniques such as multilevel anterior cervical discectomy and fusion (mACDF), anterior cervical corpectomy and fusion (ACCF), and hybrid decompression and fusion (HDF) have been proved to be reliable and effective in spinal cord decompression, and sagittal alignment restoration and maintenance thus achieved a good clinical outcome. To increase the stability of cervical vertebrae and the fusion rate of bone graft after surgery, anterior cervical titanium plate fixation is widely used. Nevertheless, the anterior titanium plate protrudes from the anterior margin of the cervical vertebral body, causing relatively strong friction with soft tissue in the anterior cervical region. As a result, complications such as foreign body sensation in the anterior cervical region, dysphagia, and esophageal injury have been found after long-term follow-up [13–15]. Meanwhile, some researchers argue that the use of anterior cervical titanium plate increases the incidence of adjacent segment degeneration (ASD) [16–18].

To prevent complications associated with anterior cervical titanium plates and maintain the benefits of interbody cages with anterior plating system, a new zero-profile, stand-alone device (Fidji cervical cage, Abbott Spine, Bordeaux, France) has been designed and used clinically [19]. In recent years, we performed mACDF using Fidji cervical cages alone (mACDF-CA) for the treatment of MCSM. In these studies, we found that mACDF-CA was associated with shorter operation time, less blood loss and cost of index surgery, and lower dysphagia incidence, and satisfactory results were achieved in preliminary clinical applications [8, 9, 20]. Despite these findings, biomechanical studies assessing anterior techniques for the treatment of MCSM appear only rarely in the literature, and no one compares mACDF-CA to other anterior techniques in multilevel constructs. A biomechanical study using finite element (FE) analysis can help to elucidate the complex biomechanical properties of the cervical spine, including stresses, strains, and loads under different conditions [21–23]. This study was a biomechanical comparative analysis of four anterior cervical techniques based on FE model. The biomechanical characteristics of the intervertebral discs at the adjacent segments and internal fixation were analyzed to provide scientific experimental evidence for surgical treatment of MCSM.

## Methods

### Development of FE model (C2-C7)

A 3-dimensional FE model of a normal C2-C7 segment was created in this study. Geometric details of the human cervical spine (C2-C7) were obtained from a high-resolution computed tomographic scan of a healthy Chinese male volunteer (age, 30 yrs.; height, 182 cm; weight, 76 kg) in our simulation. This study was approved by the medical ethics committee of First Affiliated Hospital of Dalian Medical University (PJ-KS-KY-2020-55). All procedures were followed in accordance with relevant guidelines. The subject’s skull and cervical spine were scanned using a CT scanner (Brilliance 64, Philips Electronics, Netherlands). The final CT images had a resolution of 0.54 mm × 0.54 mm and the slice interval of 0.625 mm.

**Fig. 1:**
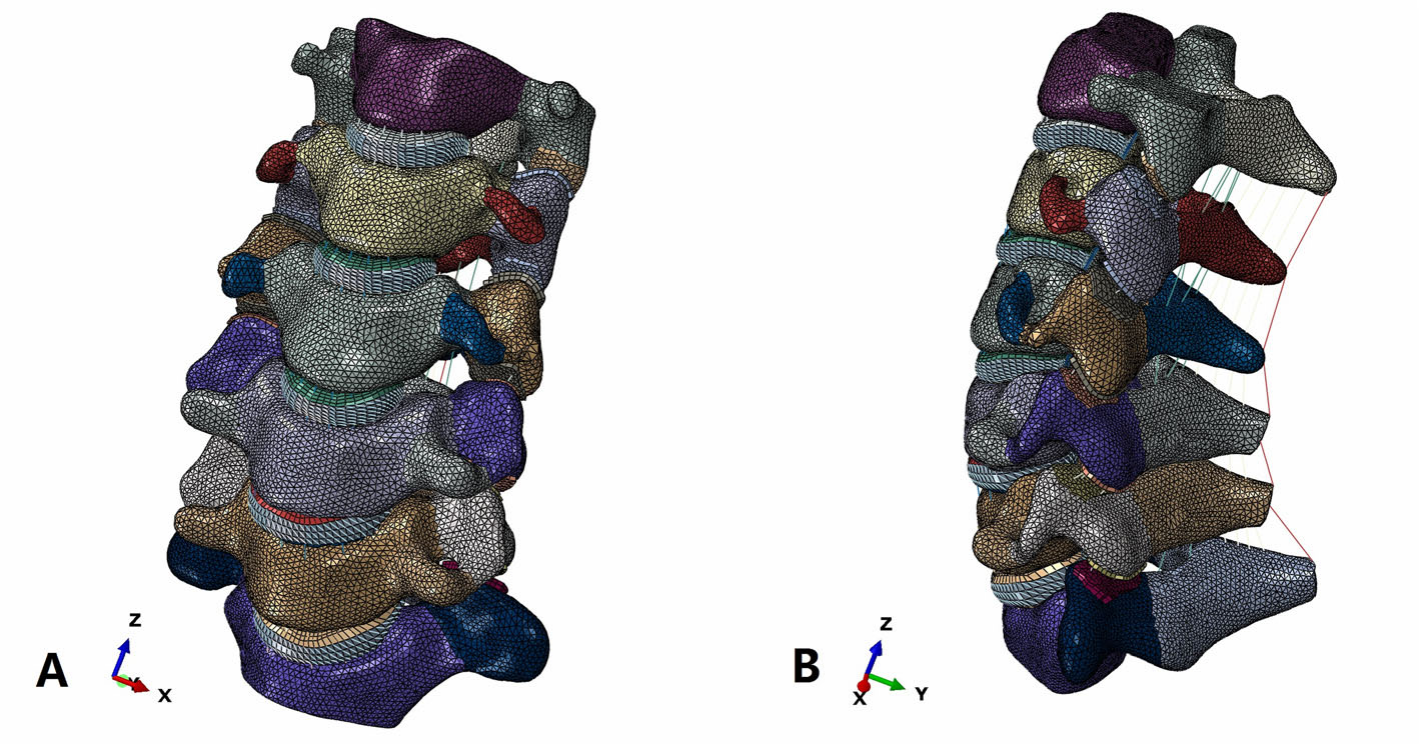
A three-dimensional finite element model of intact cervical vertebra (C2–C7). **a** Front view, **b** Lateral view

Within the software Mimics 17.0 (Materialise Inc., Leuven, Belgium), these images were segmented and translated to various 3D solid volumes of all vertebrae. Then, the solid volumes were created to fill the spaces between the vertebrae to create intervertebral discs. The final constructs were exported as STL format files. The solid volume was then, respectively, imported into the software Geomagic Studio 12.0 (Geomagic Inc., USA), in which it was converted into a non-uniform rational B-spline surface geometry structure. The model components included cortical bone, cancellous bone, bony posterior elements, annulus fibrosus, nucleus pulposus, posterior facets, end plates, anterior longitudinal ligament, posterior longitudinal ligament, ligamentum flavum, interspinous ligament, and capsular ligaments (Fig. 1). To reduce the resources required for creating a mesh of the complex spinal geometry, ABAQUS 6.13 (Abaqus Inc., USA) was used to generate a tetrahedral mesh on the vertebrae and a hexahedral mesh on the discs. The material properties were assumed to be homogeneous and isotropic according to the published literature [24–29]. The annular fibers embedded in the ground substance were assembled in a crisscross manner. The facet joint was created as a nonlinear three-dimensional contact problem using surface-to-surface contact elements. Surface to surface contact algorithm is used in defining facet joint interaction and friction coefficient was assumed to be 0.1. The initial material properties were based on previous studies as shown in Table 1.

**Table 1:** Material properties and element types of the spine soft tissues and hard tissues used in the finite element model

Component	Element type	Young’s modulus (***MPa***)	Cross-section (***mm***^**2**^)	Poisson’s ratio
**Bone**				
**Cortical bone**	Shell elements	10,000	–	0.29
**Cancellous bone**	3-D solid elements (4 node)	100	–	0.29
**Disc**				
**Annulus (ground)**	3-D solid elements (8 node)	4.2	–	0.45
**Annulus (fiber)**	3-D solid elements (8 node)	450	–	0.30
**Nucleus**	3-D solid elements (8 node)	1	–	0.49
**End plate**	3-D solid elements	500	–	0.40
**Ligaments**				
**Anterior longitudinal ligament**	3-D tension truss elements	30	33	0.30
**Posterior longitudinal ligament**	3-D tension truss elements	20	33	0.30
**Ligamentum flavum**	3-D tension truss elements	5	50.1	0.30
**Capsular ligaments**	3-D tension truss elements	20	46.6	0.30
**Interspinous ligament**	3-D tension truss elements	1.5	13	0.39
**Implants**				
**PEEK cage**	3-D solid elements (4 node)	3600	–	0.30
**Titanium plate**	3-D solid elements (4 node)	120,000	–	0.30
**Titanium screw**	3-D solid elements (4 node)	120,000	–	0.30

### Validation of model

Three-dimensional surface contact elements were used for the contact and sliding effect between the articular facets. Statistical analysis was performed by applying 1.0 Nm of flexion, extension, axial rotation, and lateral bending moments with 73.6 N of axial compression superior to C2. The boundary condition was simulated by fixing the inferior surface of the C7 vertebra under constraint of different degrees of freedom. The validity of the FE model was verified by comparing the predicted data with the results reported in the literature [30–33].

### Surgery simulation

First, a graphic of the titanium plates, screws, and PEEK interbody cages was drawn using the pre-processor modeling function of the FE software, ABAQUS/CAE. Small portions of the curved surfaces and threads were removed because these were considered not to affect the mechanical performance analysis, and rough models were constructed according to the structures of the titanium plates, screws, and cages.

mACDF model: The discectomies were simulated by removing the C3/4, C4/5, and C5/6 intervertebral discs and the corresponding anterior and posterior longitudinal ligaments. After decompression, a suitably sized PEEK interbody cage (height 5.8 mm, width 14.6 mm, and length 15.5 mm) was place into each intervertebral disc space. After cages placement, a titanium plate (height 51.3 mm, width 14.5 mm, and thickness 2.3 mm) was rigidly placed on the anterior C3-C6 vertebral bodies to provide additional stability to the fusion model. Along the ends of the anterior plate, two titanium screws were placed inside both C3 and C6 vertebral bodies within 1.00-mm distance from the end plates. Unicortical screws of 16- and 18-mm length with a mean diameter of 3.0 mm were used. For all surgical models, the interfaces at the cage-endplate and screw-bone were defined as a tied contact condition to simulate a complete fusion status. ACCF model: The C3/4, C4/5, and C5/6 intervertebral discs were resected followed by corpectomy of the C4 and C5 vertebral bodies. Also, the anterior and posterior longitudinal ligament for the C3/4, C4/5, and C5/6 motion segments were excised. A bone graft (height 43.2 mm, width 14.4 mm, and length 15.2 mm) with a cross-sectional area accounting for 50% of the vertebral endplate area was placed on the midline between the C3 and C6 vertebral bodies. The anterior margin of the bone graft was set 1.0 mm mm from the anterior margin of the vertebral bodies. Similar to the surgical procedure in mACDF, ACCF model was fixed by the same anterior plate-screw system. For all surgical models, the interfaces at the bone graft-endplate and screw-bone were defined as a tied contact condition to simulate a complete fusion status.

HDF model: The C3/4, C4/5, and C5/6 intervertebral discs were resected, followed by corpectomy of the C4 vertebral body with both sides retained. Also, the anterior and posterior longitudinal ligament for the C3/4, C4/5, and C5/6 motion segments were excised. A bone graft (height 26.7 mm, width 14.4 mm, and length 15.2 mm) of the appropriate length was placed between the C3 and C5 vertebral bodies, 1.0 mm from the anterior margin of the vertebral bodies and centered between the left and right vertebral bodies. Similar to the surgical procedure in mACDF, the same cage was placed in the C5/6 intervertebral disc space. After cage placement, a titanium plate (height 34.6 mm, width 14.5 mm, and thickness 2.3 mm) was rigidly placed on the anterior C3-C5 vertebral bodies. Unicortical titanium screws of 16- and 18-mm length with a mean diameter of 3.0 mm were used.

**Fig. 2:**
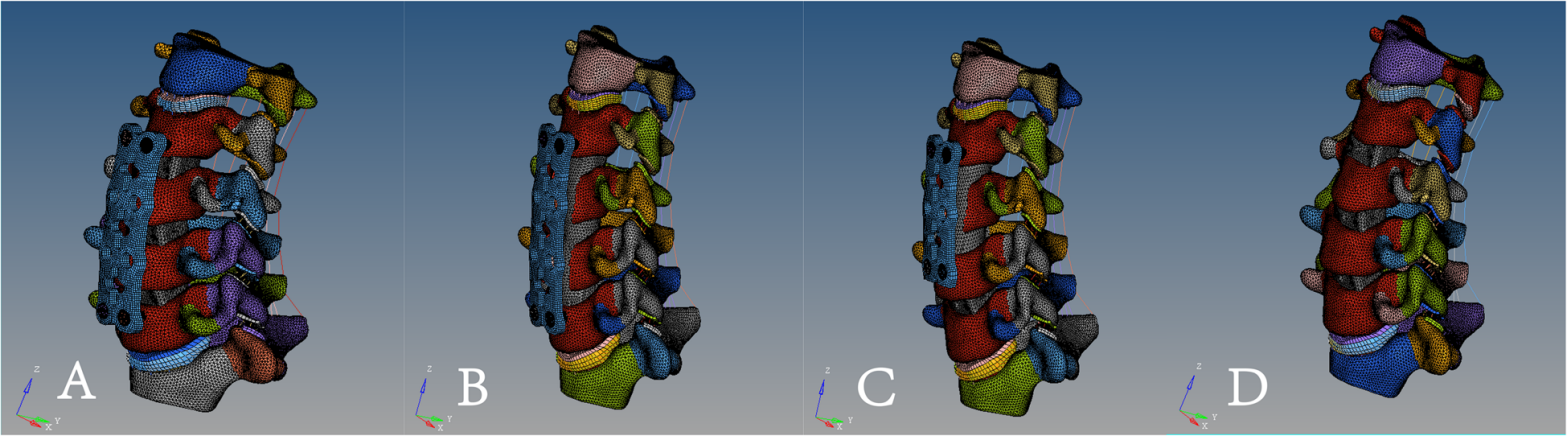
A three-dimensional finite element model of four anterior cervical techniques. **a** multilevel anterior cervical discectomy and fusion (mACDF), **b** anterior cervical corpectomy and fusion (ACCF), **c** hybrid decompression and fusion (HDF), **d** multilevel anterior cervical discectomy and fusion with cage alone (mACDF-CA)

mACDF-CA model: Similar to the surgical procedure in mACDF, the C3/4, C4/5, and C5/6 intervertebral discs and the corresponding anterior and posterior longitudinal ligaments were resected and the same cage was placed in each intervertebral disc space. This technique did not require auxiliary anterior titanium plate fixation (Fig. 2).

## Results

### Model validation

**Fig. 3:**
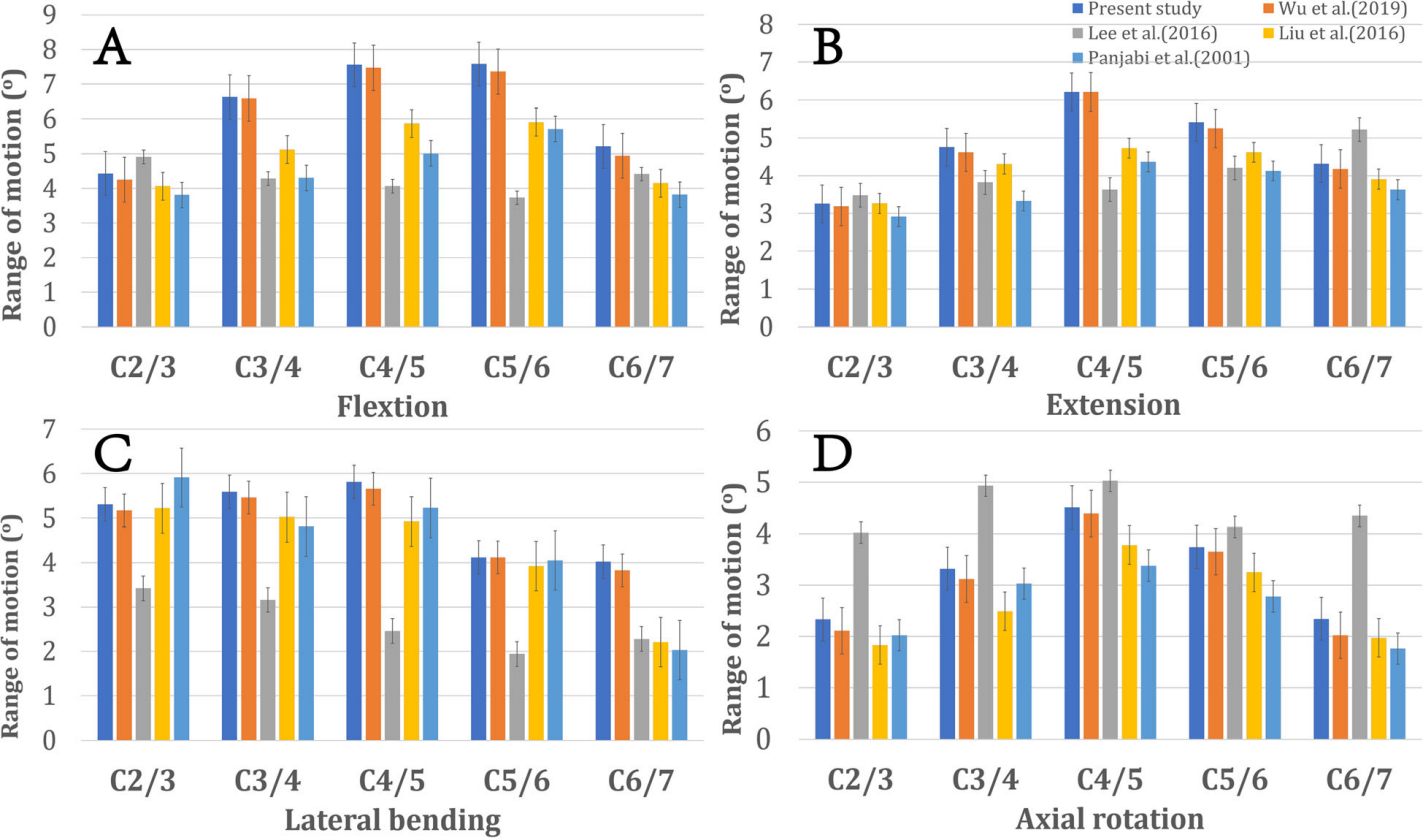
Comparison of finite element analysis results with previous published data [24–26]. **a** flexion, **b** extension, **c** lateral bending, **d** axial rotation

The comparison between in vitro data and predicted value in the FE model are shown in Fig. 3. The ROMs of the intact model at C2/3, C3/4, C4/5, C5/6 and C6/7 were 4.43°, 6.63°, 7.56°, 7.58° and 5.21°, respectively, in flexion; 3.26°, 4.76°, 6.21°, 5.41° and 4.32°, respectively, in extension; 5.31°, 5.59°, 5.81°, 4.12° and 4.02°, respectively, in lateral bending; and 2.33°, 3.32°, 4.51°, 3.74° and 2.34°, respectively, in axial rotation. All the predicted responses were consistent with the results of previous biomechanical and FE analysis studies [30–33].

### Stress on the C2/3 intervertebral disc

**Fig. 4:**
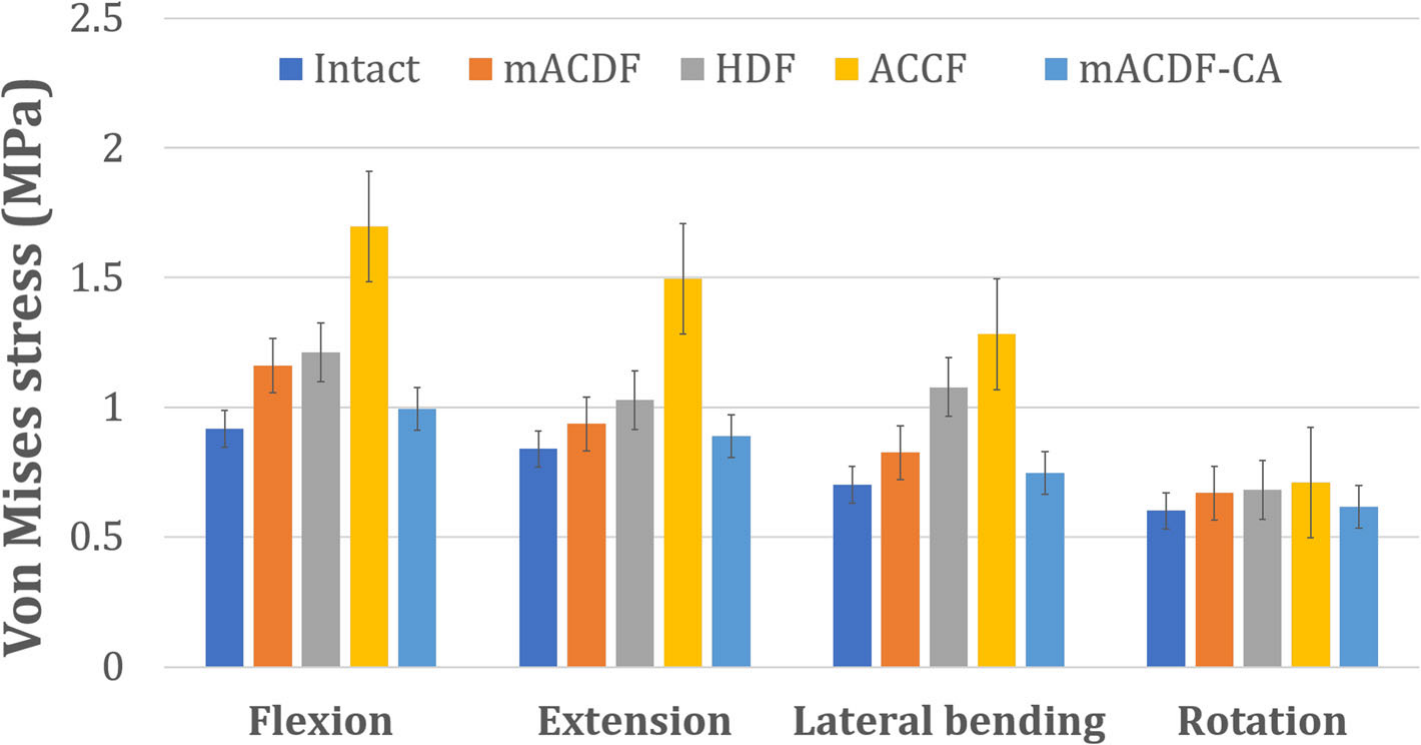
Von Mises stress at C2/3 disc in different conditions in the four models of anterior anterior cervical techniques. mACDF, multilevel anterior cervical discectomy and fusion; ACCF, anterior cervical corpectomy and fusion; HDF, hybrid decompression and fusion; mACDF-CA, multilevel anterior cervical discectomy and fusion with cage alone

**Fig. 5:**
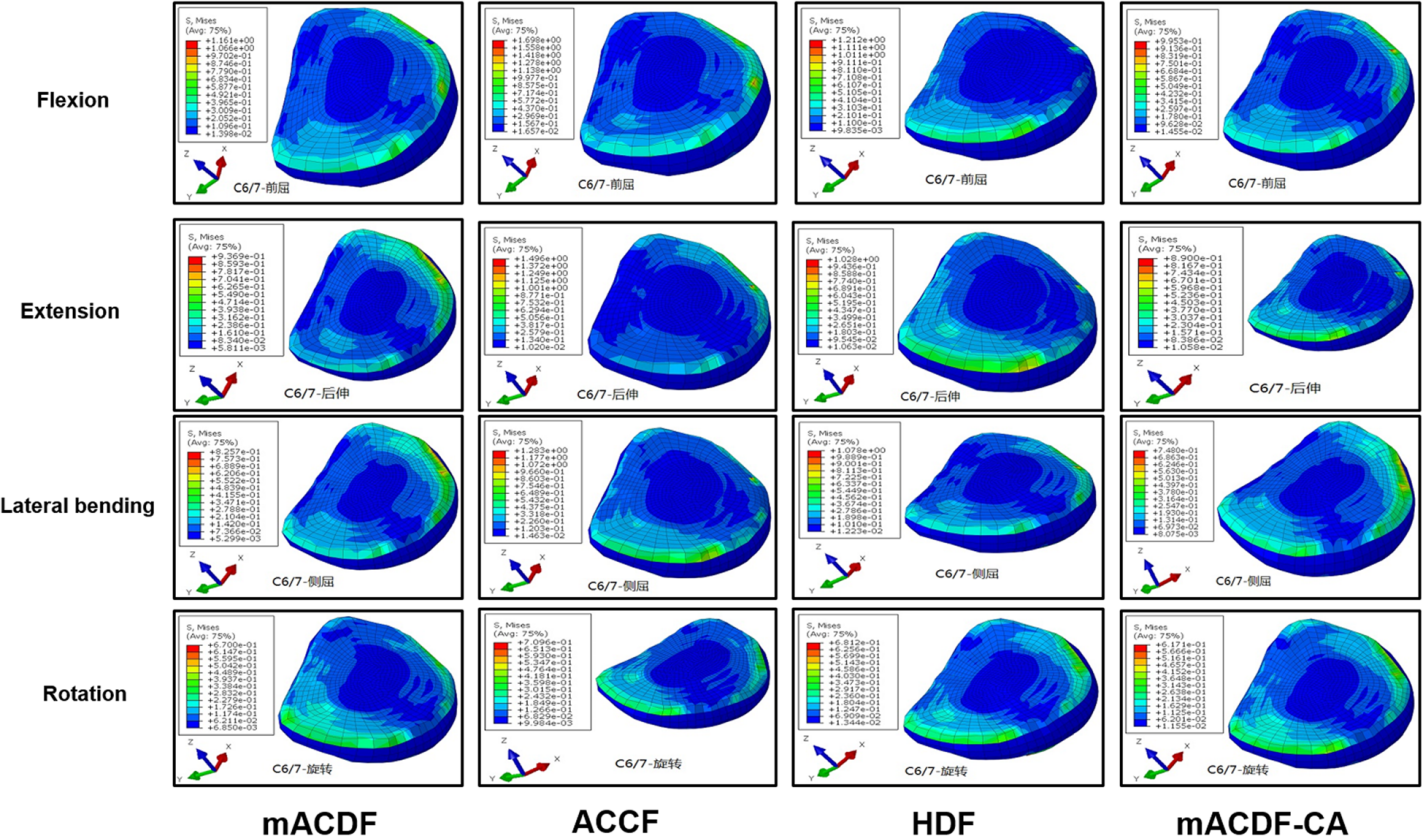
Stress distribution on the C2/3 intervertebral disc nterface in flexion, extension, lateral bending, and axial rotation

Of the four anterior cervical approaches, stress on the upper (C2/3) adjacent intervertebral disc was the lowest in the mACDF-CA group and highest in the ACCF group during flexion, extension, lateral bending, and rotation. Compared with the mACDF-CA group, the maximum von Mises stresses on the C2/3 intervertebral disc in the mACDF, ACCF, and HDF groups increased by 23, 77, and 72% during extension; by 42, 49, and 46% during lateral bending; and by 18, 104, and 105% during rotation, respectively (Figs. 4 and 5).

### Stress on the C6/7 intervertebral disc

**Fig. 6:**
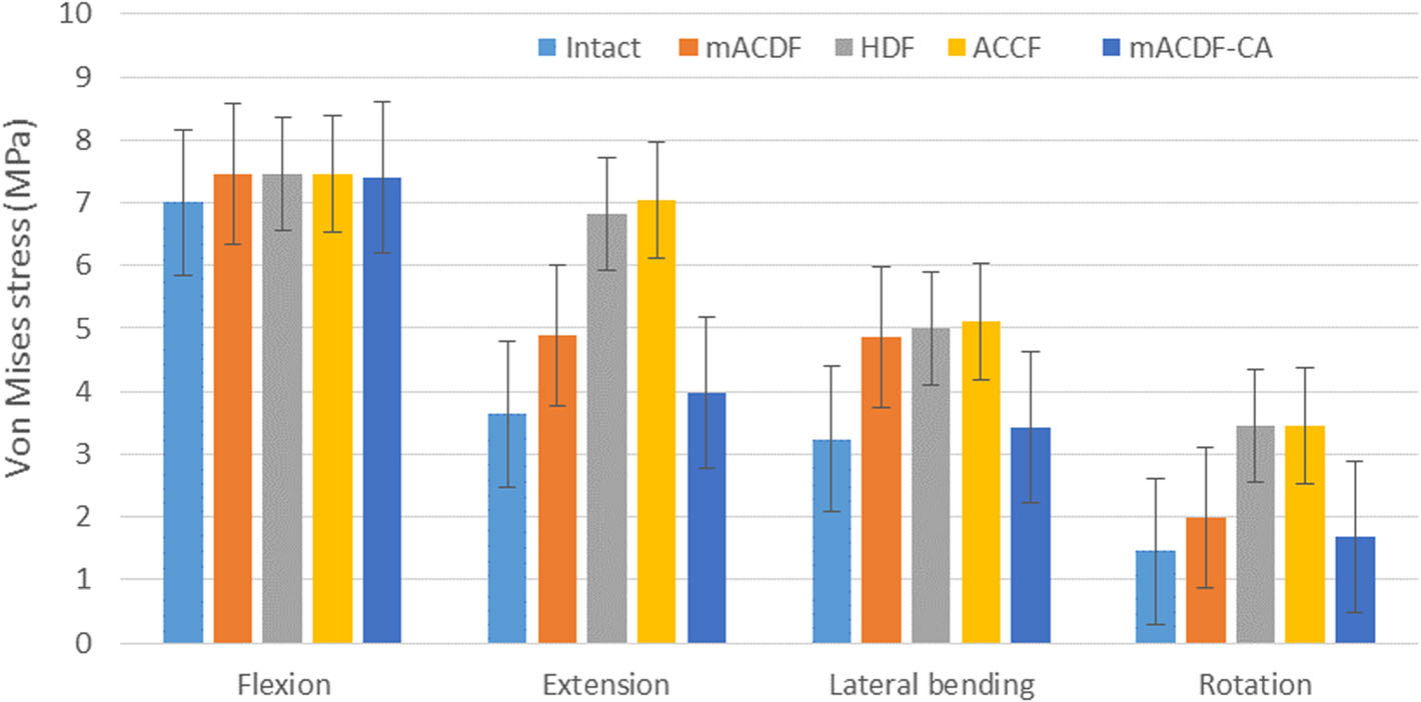
Von Mises stress at C6/7 disc in different conditions in the four models of anterior anterior cervical techniques. mACDF, multilevel anterior cervical discectomy and fusion; ACCF, anterior cervical corpectomy and fusion; HDF, hybrid decompression and fusion; mACDF-CA, multilevel anterior cervical discectomy and fusion with cage alone

**Fig. 7:**
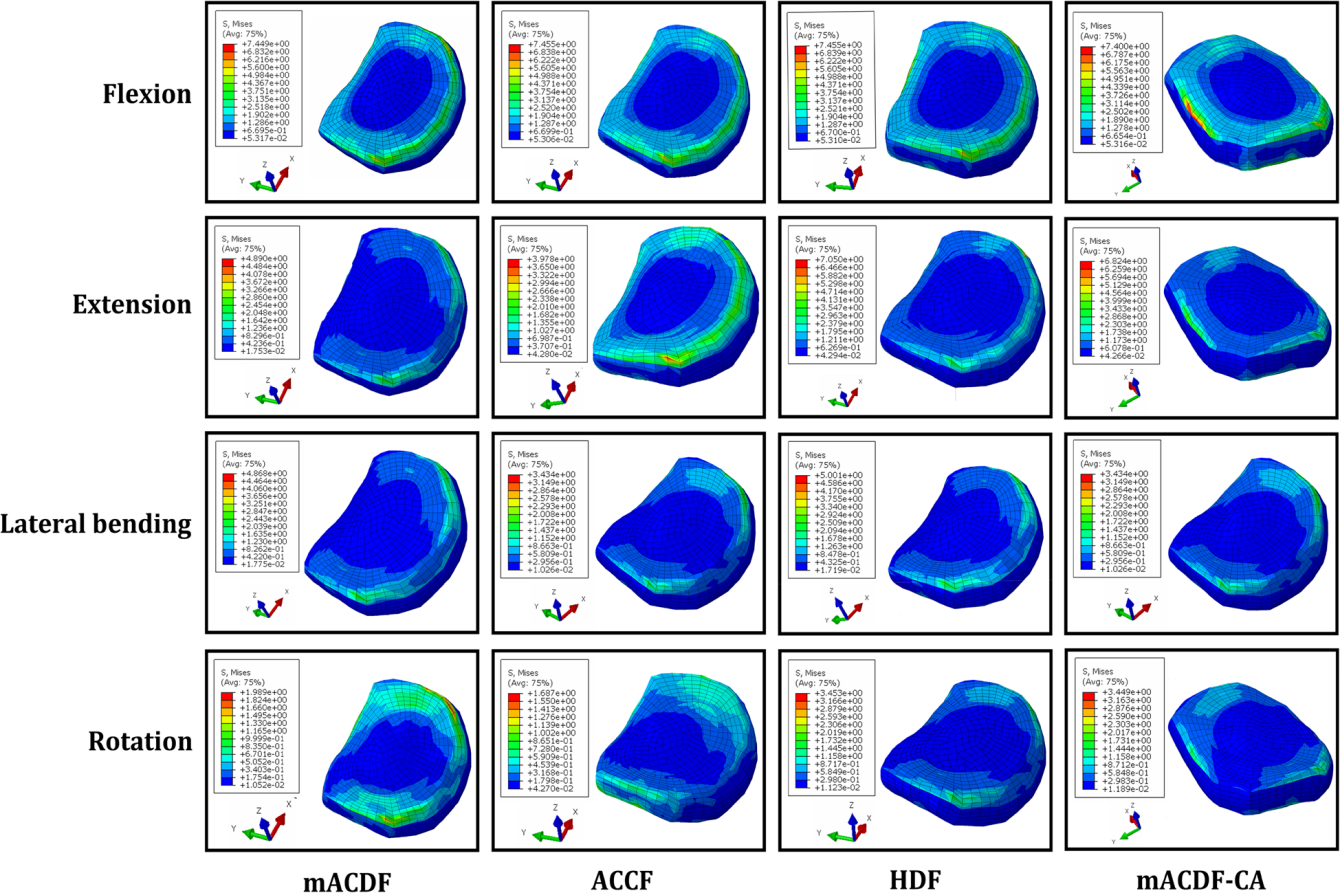
Stress distribution on the C6/7 intervertebral disc nterface in flexion, extension, lateral bending, and axial rotation

With the four anterior cervical approaches, stress on the intervertebral discs at adjacent segments was higher for the upper C2/3 than the lower C6/7 intervertebral disc during flexion, extension, lateral bending, and rotation. In all models, the mACDF-CA group had the lowest stress on the intervertebral disc, while the ACCF group had the highest stress. Compared with the mACDF-CA group, the maximum von Mises stresses on the lower (C6/7) adjacent intervertebral disc in the mACDF, ACCF, and HDF groups increased by 17, 71, and 22% during flexion; by 5, 68, and 16% during extension; by 10, 72, and 44% during lateral bending; and by 9, 15, and 10% during rotation, respectively (Figs. 6 and 7).

### Stress at the titanium plate–screw interface

**Fig. 8:**
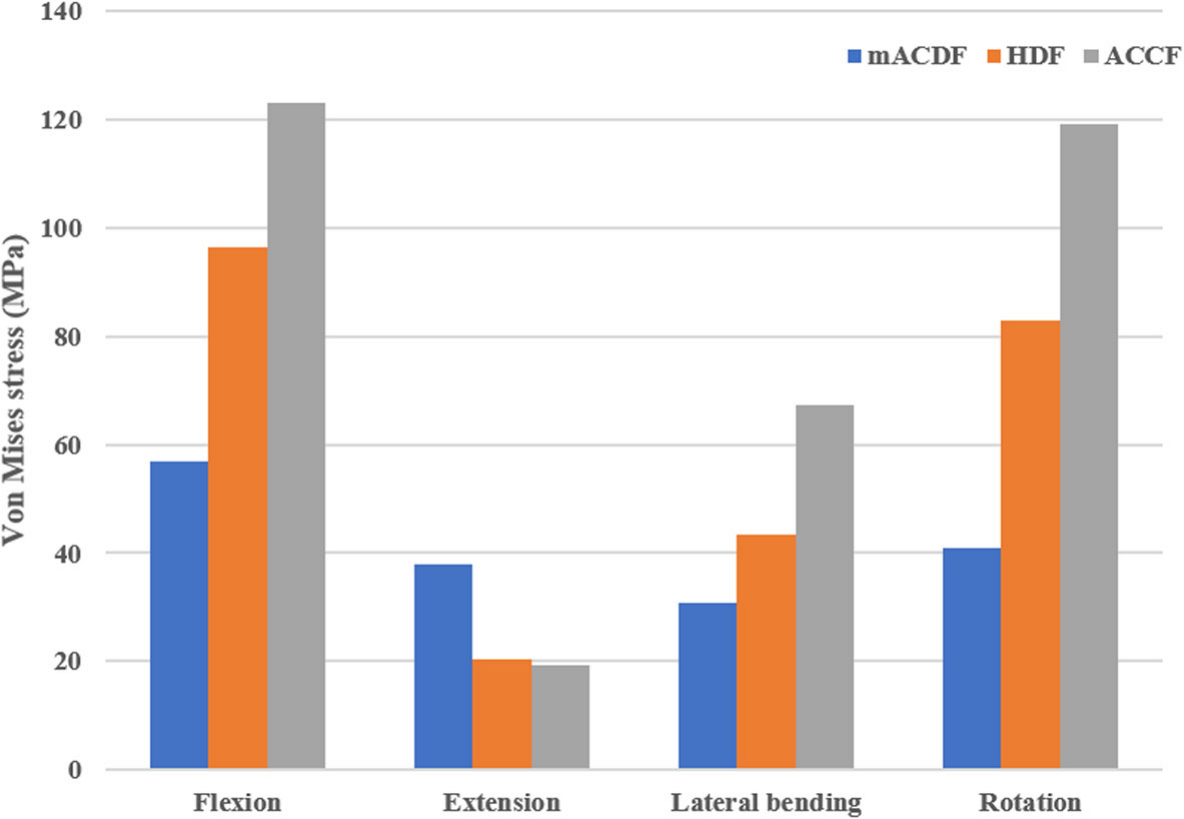
Von Mises stress at the plate-screw interface in different conditions in the three models of anterior anterior cervical techniques. mACDF, multilevel anterior cervical discectomy and fusion; ACCF, anterior cervical corpectomy and fusion; HDF, hybrid decompression and fusion

**Fig. 9:**
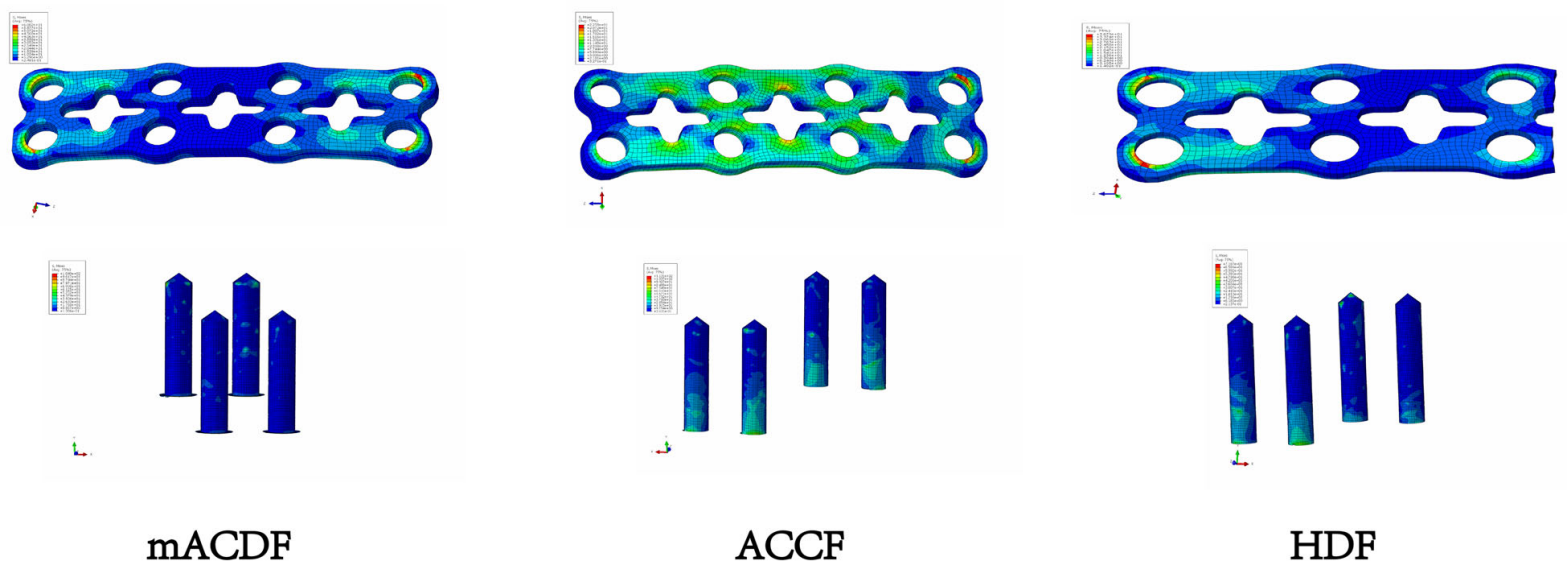
Stress distribution on the plate-screw interface in the three models of anterior anterior cervical techniques in flexion

In the three surgical models with titanium plate fixation (mACDF, ACCF, and HDF), stress at the titanium plate–screw interface was highest under flexion load and lowest under extension load. Of the three models, the ACCF group had the highest stress at the interface, while the mACDF group had the lowest stress (Figs. 8 and 9).

## Discussion

2Cervical spondylosis is a common disease in middle-aged and older people, for which surgery is a major treatment. Anterior cervical decompression and bone graft fusion is considered the standard surgical procedure for one- or two-level cervical spondylosis. However, for multilevel (three or more level) cervical spondylosis, controversy remains over the surgical approach [3–10]. As the etiological factors of MCSM usually arise from anterior degenerated intervertebral discs and osteophytes, it is challenging to remove the anterior compressive material through a simple posterior surgery, which cannot achieve effective decompression. Furthermore, posterior surgery cannot fully restore the physiological curvature of the cervical spine. Therefore, many researchers choose anterior surgery rather than posterior surgery [34]. More often than not, multiple levels are involved and complicate the surgical management. Anterior, posterior, and circumferential procedures have all been advocated. Even when the discussion is limited to anterior procedures, there is no agreement as to which reconstruction technique is best after multilevel anterior cervical decompression [1, 3, 4, 8, 9, 11, 16, 34–40].

Anterior surgical approaches mainly include ACCF, ACDF, and HDF. Among the several anterior surgical approaches for MCSM, each has advantages and disadvantages [1–4, 8, 9, 11, 16, 34–45]. In conventional ACDF surgery, osteophytes and degenerated intervertebral discs are removed from the posterior upper and lower margins of the vertebral body through an intervertebral space approach. This approach effectively removes the direct compression factors and provides good stability as well as multipoint expansion for better recovery and preservation of the physiological curvature of the cervical spine. Therefore, this approach is especially suitable for patients with straight or kyphotic curvature of the cervical spine [11, 35, 46]. However, this surgery involves a long operation time, has a limited field of view, and requires high surgical skills, making it difficult to ensure complete decompression in most cases [13–15].

ACCF involves long-segment decompression with slotting followed by long titanium mesh or autogenous bone grafting. The advantage of this approach is that it can be performed under direct vision, with a wider intraoperative view and larger operative field, and that it allows more extensive and thorough decompression [10, 37, 39, 41–43, 47, 48]. The resected vertebral body can be used as a bone autograft, thus preventing the risk associated with bone allografts and complications, such as pain in the bone removal area. Moreover, the size of the graft–host bone interface requiring postoperative healing is reduced compared with that in ACDF, which is beneficial to improve the fusion rate after surgery. The disadvantage of ACCF is that it results in considerable damage to the structural stability of the anterior and middle columns [1, 4, 8, 16, 35, 49]. Furthermore, iliac bone autografts collapse easily, and may become displaced or form a false joint. Long titanium mesh or fibular autografts are not conducive to restoring physiological lordosis of the cervical spine. In addition, owing to the multiple fixed segments and long moment arm, the monocortically-fixed screws at both ends of the titanium plate bear considerable stress, which may lead to postoperative complications, such as loosening and displacement. If the implanted bone is too long, surgical difficulty increases. Moreover, the fusion rate of long-segment bone grafting is substantially reduced, and the complication rate increases. ACCF surgery is mainly suitable for cases with lesions extending to the posterior vertebral body, extensive and severe osteophyte formation and vertebral body deformity in the anterior spinal cord, and contiguous stenosis of adjacent intervertebral spaces causing spinal cord compression.

Another anterior surgical approach is HDF, namely ACCF combined with ACDF [3, 12]. Generally, the most severely compressed vertebral body is removed in HDF, and discectomy is performed only at the less compressed sites, which reduces the number of resected vertebral bodies. While achieving full decompression, this approach also reduces damage to the anterior vertebral column, which shortens the length of the bone graft, reduces the graft–host bone interface, and theoretically, lowers the probability of upper false joint formation. However, this approach is also associated with loss of cervical lordosis and bone graft–titanium plant-related complications.

Anterior titanium plating is required with conventional mACDF, ACCF, and HDF to treat MCSM. The application of an anterior locking titanium plate can effectively improve the stability and firmness of the fused cervical spine and greatly increase the fusion rate. In addition, using a plate prevents loss of intervertebral height, while the physiological curvature of the cervical spine is maintained, to some extent. However, with increasing plate length, stress at the plate–screw interface increases correspondingly, which increases the risk of implant loosening, displacement, and fracture. Moreover, following the application of a long-segment titanium plate, patients are prone to foreign body sensation, dysphagia, and even esophageal fistula, while the incidence of ASD is also increased [13–18]. In the present study, our biomechanical results showed that among the three surgical models involving titanium plate fixation (mACDF, ACCF, and HDF groups), the ACCF group had the highest stress at the plate–screw interface, the HDF group had higher stress than the mACDF group, and the mACDF group had the lowest stress. These results revealed that the risk of titanium plate or screw loosening, displacement, and fracture was the highest following ACCF, which is similar to clinical results. Furthermore, we found that stress in the intervertebral fusion cage also differed substantially between the mACDF and mACDF-CA groups. The mACDF-CA group showed markedly higher stress than the mACDF group, which may indicate a higher risk of fusion cage subsidence in the mACDF-CA group compared with the mACDF group. However, this speculation must be verified with long-term follow-up results from controlled clinical trials with large sample sizes.

To overcome the problems associated with anterior cervical titanium plating, a novel intervertebral fusion system that integrates support, fixation, and fusion; does not protrude from the anterior margin of the vertebral body; and effectively reduces surgical complications has been designed and applied clinically. This system can be independently applied in ACDF surgery without requiring anterior titanium plate fixation. The system highlights establishing cervical stability while minimizing interference with adjacent tissues by the implant, and considerably reduces the incidence and severity of associated complications after surgery. The system has achieved satisfactory results in its preliminary clinical applications [9, 19, 20, 50–54]. The currently available self-stabilizing zero-profile anterior cervical interbody fusion and internal fixation systems are the Zero-P system (Synthes, Switzerland) and the Fidji cage system (Zimmer, France). Strong evidence from basic research and clinical use have demonstrated the effectiveness of these systems [9, 19, 20, 50–55].

ASD has always been a potential long-term complication following anterior cervical fusion, and the incidence of ASD is even higher following long-segment fusion, which has attracted increasing attention. The incidence of ASD within 10 years after primary anterior cervical surgery is 25%, and more than 15% of patients require secondary surgery owing to ASD [16–18, 56–59]. The mechanisms underpinning the development of ASD are still unclear, and the widely accepted mechanisms are local biomechanical changes in the cervical spine and natural degeneration of adjacent segments. Other risk factors are advanced age, multilevel fusion, postoperative cervical alignment change, an excessively long titanium plate, surgical injury to the adjacent intervertebral discs, and preoperative degeneration of adjacent segments. Controversy continues regarding whether differences exist in the impact of anterior cervical fusion on adjacent segments. Based on a 2-year follow-up of 218 patients undergoing single- or two-level ACCF, Park et al. [56] found that the incidence of ASD in the upper adjacent segment was markedly higher than that in the lower adjacent segment following ACCF (58% vs. 28%, respectively). In addition, Yang et al. [57] conducted a 5-year follow-up of 370 patients who underwent anterior cervical fusion without titanium plate implantation, and found that the incidence of ASD in the upper adjacent segment was considerably higher than that in the lower adjacent segment (5% vs. 1%, respectively). However, in a 5.6-year follow-up study, Koller et al. [58] found no significant difference in the incidence of ASD between the upper and lower segments adjacent to the fused segment following anterior cervical fusion (41.2% vs. 50.0%, respectively). Similarly, Goffin et al. [59] followed 25 patients for an average of 7 years and found that 24% of the patients had ASD in the upper adjacent segment, while 28% had ASD in the lower adjacent segment; the difference between the two groups was not significant. In the present study, we found that with all four anterior cervical approaches, stress on the intervertebral discs at the adjacent segments was always higher for the upper disc compared with the lower disc under different conditions (flexion, extension, lateral bending, and rotation). This result suggests that after fusion, the upper adjacent segment was subjected to higher stress, which may accelerate dehydration and degeneration of the adjacent intervertebral disc, leading easily to ASD at this segment. A plausible reason for this difference is that the lower segment has greater mobility, which is conducive to stress load sharing; however, this hypothesis requires further clinical verification. Furthermore, we found that stress on the intervertebral discs at the adjacent segments was lowest in the mACDF-CA group and highest in the ACCF group. Biomechanically, this result revealed that mACDF-CA had the least impact on the adjacent segments compared with mACDF, HDF, and ACCF. However, whether mACDF-CA effectively prevents ASD remains to be clinically verified.

## Conclusions

In summary, our biomechanical analysis indicated that among the four surgical approaches to anterior cervical fusion and internal fixation to treat MCSM, mACDF-CA had the least impact on the biomechanics of adjacent segments, and theoretically could reduce the incidence of ASD. However, this approach is associated with increased risk of fusion cage subsidence. In addition, stress at the titanium plate–screw interface was highest in the ACCF group and lowest in the mACDF group, which indicates the highest risk of titanium plate screw loosening, displacement, and fracture after ACCF. This study presented biomechanical evidence for the surgical treatment of MCSM and also provided strategies for preventing or reducing associated complications. However, further experiments and prospective clinical trials must be conducted to verify our findings.

## Data Availability

Summarized data have been presented in this manuscript. The raw data for this study are located and protected at First Affiliated Hospital of Dalian Medical University. Sharing of the raw data is not suggested, because a secondary analysis is planned.

## Abbreviations

ACDF: Anterior cervical discectomy and fusion
ALL: Anterior longitudinal ligament
ASD: Adjacent segment disease
CSM: Cervical spondylotic myelopathy
CT: Computed tomography
FE: Finite element
HDF: Hybrid decompression and fusion
mACDF: Multilevel anterior cervical discectomy and fusion
mACDF-CA: Multilevel anterior cervical discectomy and fusion with cage alone
MCSM: Multilevel cervical spondylotic myelopathy
PLL: Posterior longitudinal ligament
ROM: Range of motion
